# Optimal Resource Allocation for Uplink Data Collection in Nonorthogonal Multiple Access Networks

**DOI:** 10.3390/s18082542

**Published:** 2018-08-03

**Authors:** Yuan Wu, Cheng Zhang, Kejie Ni, Liping Qian, Liang Huang, Wei Zhu

**Affiliations:** 1 College of Information Engineering, Zhejiang University of Technology, Hangzhou 310023, China; 2111703005@zjut.edu.cn (C.Z.); 2111703062@zjut.edu.cn (K.N.); lpqian@zjut.edu.cn (L.Q.); lianghuang@zjut.edu.cn (L.H.); weizhu@zjut.edu.cn (W.Z.); 2 State Key Laboratory of Integrated Services Networks, Xidian University, Xi’an 710071, China

**Keywords:** nonorthogonal multiple access, radio resource management, uplink data collection, optimization

## Abstract

Accommodating massive connectivity for Internet of Things (IoT) applications is considered an important goal of future 5G cellular systems. Nonorthogonal multiple access (NOMA), which enables a group of mobile users to simultaneously share the same spectrum channel for transmission, provides an efficient approach to achieve the goals of spectrum-efficient data delivery. In this paper, we consider an uplink transmission in a sensor network in which a group of smart terminals (e.g., sensors) use NOMA to send their collected data to an access point. We aim to minimize the total radio resource consumption cost, including the cost for the channel usage and the cost for all senors’ energy consumption to allow the sensors to complete their data delivery requirements. Specifically, we formulate a joint optimization of the decoding-order, transmit-power and time allocations to study this problem and propose an efficient algorithm to find the optimal solution. Numerical results are provided to validate our proposed algorithm and the performance advantage of our proposed joint optimization for the uplink data collection via NOMA transmission.

## 1. Introduction

Accommodating massive connectivity for machine-type communications is considered one of the crucial goals of future fifth generation (5G) cellular systems. Nonorthogonal multiple access (NOMA), which enables a group of smart terminals (STs) (e.g., the Internet of Things devices) to share the same frequency channel simultaneously and further utilizes successive interference cancellation (SIC) to reduce the co-channel interference, has provided a promising solution towards achieving this important goal [1,2,3,4]. Compared with conventional orthogonal multiple access (OMA), which allocates orthogonal resource blocks to smart terminals and thus suffers from the limited number of available resource blocks, NOMA has been envisioned to facilitate massive connectivity for a large number of STs’ data deliveries in a spectrum-efficient manner. Meanwhile, NOMA also plays a vital role in enabling ultra-high throughput and low-latency transmission in 5G systems.

However, due to allowing co-channel interference, reaping the benefits of NOMA necessitates careful resource management which has thus motivated significant research effort in recent years. Many studies have been devoted to investigating the power allocation for NOMA. In reference [5], Zhu et al. investigated the optimal power allocation with a given channel assignment under different performance criteria (e.g., the max-min fairness and the weighted sum rate maximization). In reference [6], a two-user power allocation scheme was proposed to maximize the sum rate within Quality of Service (QoS) constraints. Driven by the growing demand for green communication [7,8,9,10], in reference [11], Zhang et al. proposed a power allocation scheme for maximizing the energy efficiency with QoS constraints for users. In addition to power allocation alone, many studies have investigated joint power allocation and channel allocation for NOMA [12,13,14]. For instance, in reference [12], Lei et al. proposed joint optimization of channel allocation and power allocation for downlink NOMA for maximizing the total throughput. In reference [13], Fang et al. jointly optimized the subchannel assignment and power allocation to maximize the energy efficiency for the downlink NOMA network. In reference [14], joint sub-channel allocation, power allocation and user scheduling for downlink NOMA throughput maximization was proposed.

Meanwhile, exploiting NOMA for different paradigms and applications has also attracted lots of interest [15,16,17,18,19]. For instance, in [15], Elbamby et al. studied resource allocation for the in-band full duplex-enabled NOMA networks. In reference [16], Sun et al. studied the MIMO NOMA system to improve energy efficiency. In reference [17], Wu et al. studied the optimal power allocation and traffic scheduling for relay-assisted networks via NOMA transmission. In reference [18], NOMA-enabled mobile edge computing was proposed for 5G systems. In reference [19], energy-efficient NOMA-enabled traffic offloading via dual-connectivity was proposed. Recently, there has been a growing interest in exploiting NOMA for IoT applications [20,21,22]. For instance, in reference [20], Shirvanimoghaddam et al. discussed the exploitation of a massive NOMA technique as a solution to support a massive number of IoT devices in cellular networks and identified its challenges. In reference [21], by exploiting the NOMA transmission, Mostafa et al. proposed a joint subcarrier and transmission power allocation problem to maximize the number of served IoT devices while satisfying the quality of service and transmission power requirements.

In this work, we consider that a group of smart terminals (e.g., sensors) are monitoring the environment and using NOMA to send their collected data to an access point (AP) simultaneously (e.g., the real-time data collection from smart meters in the emerging smart grids [23]). Our detailed contributions can be summarized as follows:

- Taking into account the different data delivery requirements of the smart terminals (STs) and their respective channel power gains to the AP, we formulate joint optimization of the SIC decoding-order for the channel-usage time and power allocations for the STs (according to [2,3] and Chapter 6 in [24], an arbitrary SIC decoding-order is viable for the uplink NOMA transmission. However, different SIC decoding-orders will yield different throughputs and power consumption levels for different STs). Our objective is to minimize the total cost for resource consumption, which includes the channel usage and the total energy consumption of all STs, for finishing all STs’ data delivery requirements.
- Despite the non-convexity of the formulated joint optimization, we propose an efficient algorithm to compute the optimal solution. Specifically, we exploit the layered structure of the problem and firstly solve the optimal time and power allocation under a given SIC decoding-order. In particular, after executing a series of equivalent transformations, we identify the convexity of the subproblem regarding the joint time and power allocations and thus find the corresponding optimal solution efficiently. By exploiting the optimal time and power allocations under each given SIC decoding-order, we further propose an efficient iterative algorithm to find the optimal SIC decoding-order.
- Extensive numerical evaluations are presented to verify the effectiveness of our proposed algorithms. Meanwhile, we also present the results of a validation of the advantages of our proposed optimal NOMA-enabled data collection scheme in comparison with using the conventional orthogonal multiple access scheme.

The reminder of this paper is organized as follows. Section 2 illustrates the system model and problem formulation. In Section 3, we propose an algorithm to find the optimal time and power allocations under a given decoding-order. In Section 4, we further propose an algorithm to find the optimal decoding-order. Finally, we conclude this work in Section 5.

## 2. System Model and Problem Formulation

### 2.1. System Model

**Figure 1:**
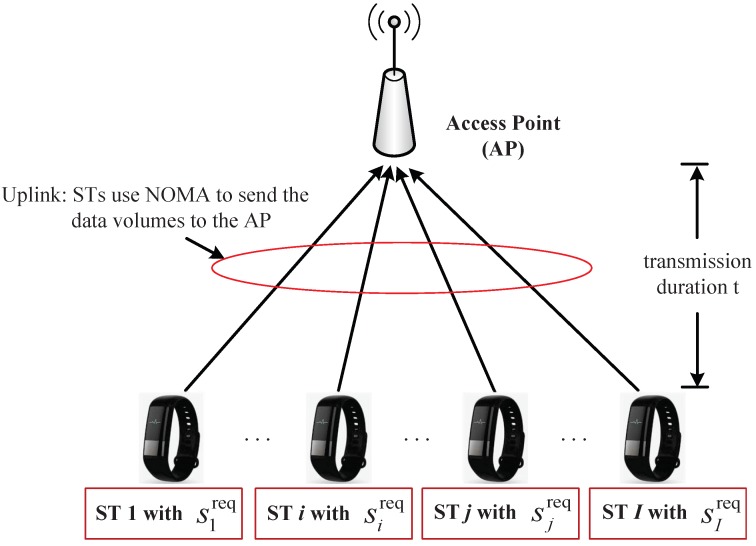
Illustration of the system model. A group of *I* STs use NOMA to send data to the AP simultaneously. Each ST *i* has a data volume of $s_{i}^{\mathrm{req}}$ to be delivered. The transmission duration of the STs’ simultaneous NOMA transmissions is denoted by *t*.

As shown in Figure 1, we consider the scenario where a group of STs I={1,2,…,I} were transmitted to an AP. Each ST, *i*, had a data volume of $s_{i}^{\mathrm{req}}$ of the collected data to be transmitted to the AP. We consider that the STs would form a NOMA-cluster to send the data volumes, ${\left\{s_{i}^{\mathrm{req}}\right\}}_{i\in I}$, to the AP over the same frequency channel simultaneously.

**Figure 2:**
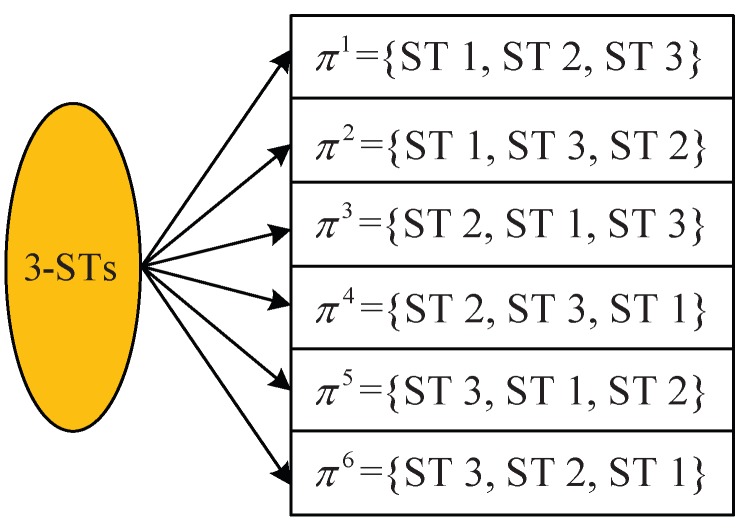
An illustration of different decoding-orders for an example of I={1,2,3} STs.

According to the multiuser capacity analysis in Chapter 6 of [24], for the uplink NOMA, an arbitrary decoding order could be viable. In other words, given the group of STs I, I! different viable decoding-orders exist (and different decoding-orders will yield different throughputs for the STs). Figure 2 illustrates an example of I={1,2,3} STs.

### 2.2. Problem Formulation under a Given Decoding-Order

To quantify each ST’s uplink throughput, we firstly consider a given decoding-order, $\pi^{m}$, where m=1,2,…,I!. Specifically, $\pi^{m}\left(i\right)$ means that the decoding-order of ST *i* under the *m*-th decoding-order, $\pi^{m}$. Given the decoding-order, $\pi^{m}$, ST *i*’s throughput to AP can be given by

(1) $$\begin{array}{c} R_{i}^{m}=W\log_{2}\left(1+\frac{p_{i}^{m}g_{iA}}{\sum_{j\in I,\pi^{m}\left(j\right)<\pi^{m}\left(i\right)}p_{j}^{m}g_{jA}+Wn_{0}}\right),\forall i\in I, \end{array}$$

where *W* denotes the uplink channel bandwidth, and $n_{0}$ denotes the spectral power density of the background noise. ${\left\{p_{i}^{m}\right\}}_{i\in I}$ denotes the profile of STs’ transmit-powers under the given decoding-ordering, *m*, and ${\left\{R_{i}^{m}\right\}}_{i\in I}$ denotes the corresponding throughput of the STs. The parameter $g_{iA}$ denotes the channel power gain from ST *i* to the AP. Similar to references [11,12,13,14], we assume a relatively static scenario in which the uplink channel power gain from each ST to the AP does not change within the scheduling duration.

For the sake of easy presentation, given the *m*-th decoding-order $\pi^{m}$, we introduce $\gamma_{i}^{m}$ as the received signal to noise plus interference ratio (SINR) for ST *i*’s uplink NOMA transmission to the AP:

(2) $$\begin{array}{c} \gamma_{i}^{m}=\frac{p_{i}^{m}g_{iA}}{\sum_{j\in I,\pi^{m}\left(j\right)<\pi^{m}\left(i\right)}p_{j}^{m}g_{jA}+Wn_{0}},\forall i\in I. \end{array}$$

As shown in Equation (2), each ST *i*’s received SINR not only depends on its own transmit-power, $p_{i}^{m}$, but also depends on the transmit-powers of those STs which are decoded before ST *i* in the *m*-th decoding-order, $\pi^{m}$. Thus, let us treat all STs’ ${\left\{\gamma_{i}^{m}\right\}}_{i\in I}$ (under the *m*-th decoding-order) as the auxiliary variables which depend on the STs’ transmit-powers. Then, based on Equation (2), we express each ST *i*’s minimum transmit-power to reach $\gamma_{i}^{m}$ as follows:

(3) $$\begin{array}{c} p_{i}^{\min,m}\left({\left\{\gamma_{j}^{m}\right\}}_{j\in I,\pi^{m}\left(j\right)\leq \pi^{m}\left(i\right)}\right)=\frac{Wn_{0}}{g_{iA}}\gamma_{i}^{m}\prod_{j\in I,\pi^{m}\left(j\right)<\pi^{m}\left(i\right)}\left(1+\gamma_{j}^{m}\right),\forall i\in I. \end{array}$$

Notice that in the uplink NOMA, all STs send their data to the AP simultaneously. Thus, we use $t^{m}$ to denote the transmission duration of the STs to send the data volumes, ${\left\{s_{i}^{\mathrm{req}}\right\}}_{i\in I}$, to the AP under the *m*-th decoding-ordering $\pi^{m}$. Correspondingly, we obtain

$$\begin{array}{c} R_{i}^{m}=\frac{s_{i}^{\mathrm{req}}}{t^{m}}=W\log_{2}\left(1+\gamma_{i}^{m}\right),\forall i\in I, \end{array}$$

which thus led to

(4) $$\begin{array}{c} \gamma_{i}^{m}=2^{\frac{s_{i}^{\mathrm{req}}}{t^{m}}\frac{1}{W}}-1,\forall i\in I. \end{array}$$

By substituting (4) into (3), we obtain each ST *i*’s minimum transmit-power, i.e.,

(5) $$\begin{array}{c} p_{i}^{\min,m}\left(t^{m}\right)=\frac{Wn_{0}}{g_{iA}}\left(2^{\frac{s_{i}^{\mathrm{req}}}{t^{m}}\frac{1}{W}}-1\right)2^{\frac{1}{t^{m}}\frac{1}{W}\sum_{j=1,\pi^{m}\left(j\right)<\pi^{m}\left(i\right)}^{I}s_{j}^{\mathrm{req}}},\forall i\in I, \end{array}$$

when using the *m*-th decoding-order, $\pi^{m}$.

Under the given *m*-th decoding-order, $\pi^{m}$, for SIC, we formulate the following optimization problem that aims to minimize the total cost of radio resource consumption when the group of STs I send their data to the AP (in Problem (P1-*m*), *m* means the *m*-th decoding-order):

$$\begin{array}{cc} (P1-m):\; & O^{m}=\min\alpha t^{m}+\beta t^{m}\sum_{i=1}^{I}p_{i}^{\min,m}\left(t^{m}\right) \end{array}$$

(6) $$\begin{array}{cc} \mathrm{subject}\;\mathrm{to}:\; & t^{m}p_{i}^{\min,m}\left(t^{m}\right)\leq E_{i}^{\max},\forall i\in I, \end{array}$$

(7) $$0\leq t^{m}\leq T^{\max}$$

$$\begin{array}{cc} \mathrm{variables}:\; & t^{m}. \end{array}$$

In the objective function of Problem (P1-*m*), the expression of $\alpha t^{m}+\beta t^{m}\sum_{i=1}^{I}p_{i}^{\min,m}\left(t^{m}\right)$ denotes the total cost for resource consumption, which includes the cost for the channel usage duration, $t^{m}$ (with the marginal cost-coefficient denoted by α) and the cost for all STs’ total energy consumption given by $t^{m}\sum_{i=1}^{I}p_{i}^{\min,m}\left(t^{m}\right)$ (with the marginal cost-coefficient denoted by β). Notice that for each ST *i*, the minimum required transmit-power, $p_{i}^{\min,m}\left(t^{m}\right)$, is given by Equation (5) before. Constraint (6) means that each ST *i*’s total energy consumption for transmission cannot exceed its maximum energy budget, denoted by $E_{i}^{\max}$. Constraint (7) means that the channel usage duration, $t^{m}$, cannot exceed the maximum duration, $T^{\max}$. Problem (P1-*m*) is a typical nonconvex optimization problem [25], and thus, no general algorithm exists that can efficiently solve Problem (P1-*m*).

In particular, in Problem (P1-*m*), we use $O^{m}$ to denote the minimum total cost. Then, to find the optimal decoding-order among the I! viable orderings for the group of I STs, we only need to solve the following problem:

(8) $$\begin{array}{cc} (P1-\mathrm{Order}):\; & \min_{m=1,2,\ldots ,I!}O^{m}. \end{array}$$

A straightforward approach to solve Problem (P1-Order) is to enumerate all I! decoding-orders; however, this is computationally prohibitive. In Section 4, we propose an efficient algorithm to solve Problem (P1-Order). As a first step, in the next section, we first provide an efficient algorithm to solve (P1-*m*) under the given *m*-th decoding order. Please notice that in this work, to focus on minimizing the overall resource consumption cost for finishing delivering STs’ required data volumes, ${\left\{s_{i}^{\mathrm{req}}\right\}}_{i\in I}$, we presume that Problem (P1-*m*) was feasible in this work. This assumption is viable when the volumes of the STs’ collected data are relatively small. As an important future direction to extend our work, we will further take into account the special case that Problem (P1-*m*) is infeasible, and propose an algorithm to check its feasibility.

## 3. Proposed Algorithm to Solve Problem (P1-*m*)

In this section, we propose an efficient algorithm to solve Problem (P1-*m*), which thus yields the minimum total cost under the given decoding-order, $\pi^{m}$. To efficiently solve this problem, we introduce a variable-change as

(9) $$\begin{array}{c} x=\frac{1}{t^{m}}. \end{array}$$

Notice that we do not use the script *m* in (9) since *x* is treated as an internal variable which can be used for an arbitrary decoding-order, $\pi^{m}$.

Using (9), we thus express ST *i*’s minimum required transmit-power as

(10) $$\begin{array}{c} p_{i}^{\min,m}\left(x\right)=\frac{Wn_{0}}{g_{iA}}\left(2^{x\frac{s_{i}^{\mathrm{req}}}{W}}-1\right)2^{x\frac{1}{W}\sum_{j\in I,\pi^{m}\left(j\right)<\pi^{m}\left(i\right)}s_{j}^{\mathrm{req}}},\forall i\in I, \end{array}$$

and Problem (P1-*m*) was equivalently transformed into (letter “E” means “equivalent”)

$$\begin{array}{cc} (P1-m-E):\; & \min\alpha \frac{1}{x}+\beta \frac{1}{x}\sum_{i\in I}p_{i}^{\min,m}\left(x\right) \end{array}$$

(11) $$\begin{array}{cc} \mathrm{subject}\;\mathrm{to}:\; & p_{i}^{\min,m}\left(x\right)\leq xE_{i}^{\max},\forall i\in I, \end{array}$$

(12) $$x\geq \frac{1}{T^{\max}}$$

$$\begin{array}{cc} \mathrm{variables}:\; & x. \end{array}$$

For the sake of easy presentation, we define an auxiliary function, $H_{i}\left(x\right)$, as

(13) $$\begin{array}{c} H_{i}\left(x\right)=\frac{Wn_{0}}{g_{iA}}\left(2^{x\frac{s_{i}^{\mathrm{req}}}{W}}-1\right)2^{x\frac{1}{W}\sum_{j\in I,\pi^{m}\left(j\right)<\pi^{m}\left(i\right)}s_{j}^{\mathrm{req}}},\forall i\in I. \end{array}$$

Recall that $H_{i}\left(x\right)$ stems from (10).

The key idea in solving Problem (P1-*m*-E) was to introduce an additional new variable, θ, which required

(14) $$\begin{array}{c} \alpha \frac{1}{x}+\beta \frac{1}{x}\sum_{i\in I}p_{i}^{\min,m}\left(x\right)\leq \theta . \end{array}$$

Thus, by using θ, we transform Problem (P1-*m*-E) into

$$\begin{array}{cc} (P2):\; & \min\theta \end{array}$$

(15) $$\begin{array}{cc} \mathrm{subject}\;\mathrm{to}:\; & \frac{\theta x-\alpha }{\beta }-\sum_{i\in I}H_{i}\left(x\right)\geq 0, \end{array}$$

(16) $$\begin{array}{c} H_{i}\left(x\right)\leq xE_{i}^{\max},\forall i\in I, \end{array}$$

(17) $$x\geq \max\{\frac{1}{T^{\max}},\frac{\alpha }{\theta }\},$$

$$\begin{array}{cc} \mathrm{variables}:\; & \theta . \end{array}$$

Notice that Problem (P2) corresponds to finding the optimal value of θ (which is denoted by $\theta^{*}$) that can meet constraints (15)–(17). The value of $\theta^{*}$ is the minimum cost of Problem (P1-*m*-E) under the *m*-th decoding-order.

The rationale to solve Problem (P2) (as well as the original Problem (P1-*m*-E)) and to determine $\theta^{*}$ is as follows. First of all, let us denote a parameterized subproblem (P2) under a given temporal value of θ:

$$\begin{array}{cc} (P2-\mathrm{Sub}):\; & V_{\theta }=\min\sum_{i\in I}H_{i}\left(x\right)-\frac{\theta x-\alpha }{\beta } \end{array}$$

(18) $$\begin{array}{cc} \mathrm{subject}\;\mathrm{to}:\; & H_{i}\left(x\right)\leq xE_{i}^{\max},\forall i\in I, \end{array}$$

(19) $$x\geq \max\{\frac{1}{T^{\max}},\frac{\alpha }{\theta }\},$$

$$\begin{array}{cc} \mathrm{variables}:\; & x. \end{array}$$

We use $V_{\theta }$ to denote the optimal objective function value of Problem (P2-Sub). In particular, if Problem (P2-Sub) output $V_{\theta }\leq 0$, then Problem (P2) is feasible under the currently given θ (which means that θ can be reduced further). On the other hand, if Problem (P2-Sub) output $V_{\theta }>0$, then Problem (P2) is infeasible under the current θ (which means that we need to increase θ). To solve the original Problem (P2), we need to further find the minimum value of θ (i.e., $\theta^{*}$) that would be able to yield the corresponding $V_{\theta }\leq 0$, i.e., we need to solve the following problem

(20) $$\begin{array}{c} (P2-\mathrm{Top}):\;\min\theta ,\;\;\mathrm{subject}\;\mathrm{to}:\;\;\;V_{\theta }\leq 0. \end{array}$$

It can be seen from Problem (P2-Sub) that the optimal output, $V_{\theta }$, decreases in θ due to the following reasons: (i) the objective function decreases in θ, and (ii) the feasible interval increases in θ. As a result, we exploit a bisection search to solve Problem (P2-Top) and find the corresponding $\theta^{*}$. We present the details of our proposed algorithm in the next subsections.

Recall that we can determine the minimum total cost for resource consumption of Problem (P1Sub-*m*) as

(21) $$\begin{array}{c} O^{m}=\theta^{*}. \end{array}$$

under the given decoding-order, $\pi^{m}$.

In the next subsection, we focus on proposing an algorithm to solve Problem (P2-Sub) under a given θ.

### 3.1. Proposed Subroutine to Solve Problem (P2-Sub)

To solve Problem (P2-Sub) under a given θ, we firstly compute the first-order derivative of $H_{i}\left(x\right)$ as follows:

(22) $$\begin{array}{c} H_{i}^{\prime }\left(x\right)=\frac{Wn_{0}}{g_{iA}}\left(2^{x\frac{s_{i}^{\mathrm{req}}}{W}}-1\right)2^{x\frac{1}{W}\sum_{j\in I,\pi^{m}\left(j\right)<\pi^{m}\left(i\right)}s_{j}^{\mathrm{req}}}\left(\ln2\right)\left(\frac{1}{W}\sum_{j=1,\pi^{m}\left(j\right)<\pi^{m}\left(i\right)}^{I}s_{j}^{\mathrm{req}}\right)\\ +\frac{Wn_{0}}{g_{iA}}2^{x\frac{1}{W}\sum_{j\in I,\pi^{m}\left(j\right)<\pi^{m}\left(i\right)}s_{j}^{\mathrm{req}}}2^{x\frac{s_{i}^{\mathrm{req}}}{W}}\left(\ln2\right)\frac{s_{i}^{\mathrm{req}}}{W}. \end{array}$$

Equation (26) shows that $H_{i}^{\prime }\left(x\right)$ increases in *x*. Based on the theory of convex optimization [25], $H_{i}\left(x\right)$ is convex with respect to *x*.

For the sake of easy presentation, we further introduce G(x) to denote the objective function of Problem (P2-Sub) as follows:

(23) $$\begin{array}{c} G\left(x\right)=\sum_{i\in I}H_{i}\left(x\right)-\frac{\theta x-\alpha }{\beta }, \end{array}$$

and correspondingly, we obtain the first-order derivative of G(x) as follows:

(24) $$\begin{array}{c} G^{\prime }\left(x\right)=\sum_{i\in I}H_{i}^{\prime }\left(x\right)-\frac{\theta }{\beta }, \end{array}$$

which, again, increases in *x* (due to $H_{i}^{\prime }\left(x\right)$ is increasing in *x*). As a result, G(x) is convex with respect to *x*.

Based on the convexity of function $H_{i}\left(x\right)$ and function G(x), we obtain the following important property.

**Proposition** **1.** *Given θ, Problem (P2-Sub) is a strict convex optimization with respect to x.*

**Proof.**  Equations (22) and (24) together lead to the objective function of Problem (P2-Sub), i.e., G(x), is a strictly convex function. Meanwhile, Equation (22) means that constraint (18) leads to a convex feasible set. As a result, according the convex optimization theory [25], Problem (P2-Sub) is a strictly convex optimization with respect to *x*, which finishes the proof. ☐

The convexity of Problem (P2-Sub) enables us to use the Karush–Kuhn–Tucker (KKT) conditions to find the optimal solution. Exploiting the KKT conditions, the following algorithm was proposed to solve Problem (P2-Sub). The details are illustrated as follows.

#### 3.1.1. Procedures to Determine the Viable Interval of *x* for Problem (P2-Sub)

Our first step was to determine the feasible interval of *x* for Problem (P2-Sub), i.e., the interval of *x* that could meet constraints (18) and (19) simultaneously.

To derive the upper-bound of *x* given by constraint (18), we notice that constraint (18) could be separated with respect to different STs. As a result, we express the upper-bound of *x* as

(25) $$\begin{array}{c} x^{\max}=\min_{i\in I}\left\{x_{i}^{\mathrm{largest}}\right\}, \end{array}$$

with the value of $x_{i}^{\mathrm{largest}}$ given by

(26) $$\begin{array}{c} x_{i}^{\mathrm{largest}}=\arg\max\left\{x\geq 0|Q_{i}\left(x\right)\geq 0\right\},\forall i\in I. \end{array}$$

Specifically, function $Q_{i}\left(x\right)$ is given by

(27) $$\begin{array}{c} Q_{i}\left(x\right)=E_{i}^{\max}x\frac{g_{iA}}{Wn_{0}}-\left(2^{x\frac{s_{i}^{\mathrm{req}}}{W}}-1\right)2^{x\frac{1}{W}\sum_{j\in I,\pi^{m}\left(j\right)<\pi^{m}\left(i\right)}s_{j}^{\mathrm{req}}},\forall i\in I, \end{array}$$

according to constraint (18). For each ST *i*, $x_{i}^{\mathrm{largest}}$ corresponds to the largest value of *x* that can ensure $Q_{i}\left(x\right)\geq 0$ (or constraint (18) is satisfied).

To find $x_{i}^{\mathrm{largest}}$, we derive the first-order derivative of $Q_{i}\left(x\right)$ as follows:

(28) $$\begin{array}{c} Q_{i}^{\prime }\left(x\right)=E_{i}^{\max}\frac{g_{iA}}{Wn_{0}}-\left(2^{x\frac{s_{i}^{\mathrm{req}}}{W}}-1\right)2^{x\frac{1}{W}\sum_{j\in I,\pi^{m}\left(j\right)<\pi^{m}\left(i\right)}s_{j}^{\mathrm{req}}}\left(\ln2\right)\left(\frac{1}{W}\sum_{j=1,\pi^{m}\left(j\right)<\pi^{m}\left(i\right)}^{I}s_{j}^{\mathrm{req}}\right)\\ -2^{x\frac{1}{W}\sum_{j\in I,\pi^{m}\left(j\right)<\pi^{m}\left(i\right)}s_{j}^{\mathrm{req}}}2^{x\frac{s_{i}^{\mathrm{req}}}{W}}\left(\ln2\right)\frac{s_{i}^{\mathrm{req}}}{W}. \end{array}$$

The above result shows that $Q_{i}^{\prime }\left(x\right)$ decreases in *x*. In other words, for each ST *i*, function $Q_{i}\left(x\right)$ is a typical unimodal function. By exploiting this property and an important feature of $Q_{i}\left(0\right)=0$, we consider the following two cases to find $x_{i}^{\mathrm{largest}}\in \left[0,\infty \right)$:

- Case-I. If $Q_{i}^{\prime }\left(0\right)\leq 0$ (i.e., $E_{i}^{\max}\frac{g_{iA}}{n_{0}}\leq s_{i}^{\mathrm{req}}\ln2$), then $x_{i}^{\mathrm{largest}}=0$.
- Case-II. If $Q_{i}^{\prime }\left(0\right)>0$ (i.e., $E_{i}^{\max}\frac{g_{iA}}{n_{0}}>s_{i}^{\mathrm{req}}\ln2$), then $x_{i}^{\mathrm{largest}}\in \left[x_{i}^{▵},\infty \right)$ can be uniquely determined by $Q_{i}\left(x_{i}^{\mathrm{largest}}\right)=0$, where the value of $x_{i}^{▵}\in \left[0,\infty \right)$ is uniquely determined by $Q_{i}^{\prime }\left(x_{i}^{▵}\right)=0$.

We explain the above two cases as follows. If $Q_{i}^{\prime }\left(0\right)\leq 0$, then $Q_{i}^{\prime }\left(x\right)$ monotonically decreases for x∈[0,∞). In this case, based on the features of $Q_{i}\left(0\right)=0$, we have $x_{i}^{\mathrm{largest}}=0$ (i.e., Case-I). On the other hand, if $Q_{i}^{\prime }\left(0\right)>0$, then a unique point exists in the interval of x∈[0,∞), such that $Q_{i}^{\prime }\left(x\right)=0$, since function $Q_{i}^{\prime }\left(x\right)$ is decreasing and we have $Q_{i}^{\prime }\left(\infty \right)<0$ according to (28). Let us denote such a point as $x_{i}^{▵}$, i.e., $Q_{i}^{\prime }\left(x_{i}^{▵}\right)=0$. Notice that, since we have $Q_{i}\left(0\right)=0$, $Q_{i}\left(x_{i}^{▵}\right)\geq 0$ always exists. As a result, a unique point exists in the interval of $\left[x_{i}^{▵},\infty \right)$, such that $Q_{i}\left(x\right)=0$. Such a point corresponds to $x_{i}^{\mathrm{largest}}$, i.e., $Q_{i}\left(x_{i}^{\mathrm{largest}}\right)=0$, which corresponds to the solution in Case-II.

The detailed procedures to find $x_{i}^{\mathrm{largest}}$ are shown in Steps 3–8 in our following proposed algorithm (i.e., Algorithm 1) to solve Problem (P2-Sub). After finding $x_{i}^{\mathrm{largest}}$ for each ST *i*, thus determined the viable interval for *x* as $x\in [\max\left\{\frac{1}{T^{\max}},\frac{\alpha }{\theta }\right\},\min_{i\in I}\left\{x_{i}^{\mathrm{largest}}\right\}]$ according to constraint (19) which corresponds to Step 12 in our Algorithm 1.

#### 3.1.2. Procedures to Determine $V_{\theta }$ for Problem (P2-Sub)

After deriving the viable interval $x\in [\max\left\{\frac{1}{T^{\max}},\frac{\alpha }{\theta }\right\},\min_{i\in I}\left\{x_{i}^{\mathrm{largest}}\right\}]$, we then continue to solve Problem (P2-Sub) and obtain $V_{\theta }$. To this end, we exploit the convexity of function G(x) (i.e., its first order derivative $G^{\prime }\left(x\right)$ in (24) is decreasing), and used the KKT conditions to find the minimum value of the objective function, i.e., $V_{\theta }$. To this end, we need to consider the following two cases:

- If $G^{\prime }\left(0\right)=n_{0}\ln2\sum_{i\in I}\frac{s_{i}^{\mathrm{req}}}{g_{iA}}-\frac{\theta }{\beta }\;<0$, then a unique point, μ∈[0,∞), exists such that $G^{\prime }\left(\mu \right)=0$.
- If $G^{\prime }\left(0\right)=n_{0}\ln2\sum_{i\in I}\frac{s_{i}^{\mathrm{req}}}{g_{iA}}-\frac{\theta }{\beta }\geq 0$, no such μ∈[0,∞) exists such that $G^{\prime }\left(\mu \right)=0$, meaning that G(x) is increasing for x∈[0,∞).

Step 16 to Step 30 in our Algorithm 1 correspond to the operations of the above two cases.

**Algorithm 1** To solve Problem (P2-Sub) and output $V_{\theta }$.
1:**Initialization:** Set i=1, and set a sufficiently large upper-bound $x^{\mathrm{uppbound}}$ for the value of *x*.2:
**while** i≤I **do**
3: **if** $E_{i}^{\max}\frac{g_{iA}}{n_{0}}>s_{i}^{\mathrm{req}}\ln2$ **then**4:  Firstly, use the bisection-search method to find $x_{i}^{▵}\in \left[0,x^{\mathrm{uppbound}}\right]$, such that $Q_{i}^{\prime }\left(x_{i}^{▵}\right)=0$.5:  Secondly, use the bisection-search method to find $x_{i}^{\mathrm{largest}}\in \left[x_{i}^{▵},x^{\mathrm{uppbound}}\right]$ such that $Q_{i}\left(x_{i}^{\mathrm{largest}}\right)=0$.6: **else**7:  Set $x_{i}^{\mathrm{largest}}=0$.8: **end if**9: Update i=i+1.10:
**end while**
11:
**if** $\max\left\{\frac{1}{T^{\max}},\frac{\alpha }{\theta }\right\}<\min_{i\in I}\left\{x_{i}^{\mathrm{largest}}\right\}$ **then**
12: Set the viable interval for *x* as $x\in [\max\left\{\frac{1}{T^{\max}},\frac{\alpha }{\theta }\right\},\min_{i\in I}\left\{x_{i}^{\mathrm{largest}}\right\}]$.13:
**else**
14: Output that Problem (P2-Sub) is infeasible.15:
**end if**
16:
**if** $n_{0}\ln2\sum_{i\in I}\frac{s_{i}^{\mathrm{req}}}{g_{iA}}<\frac{\theta }{\beta }$ **then**
17: Use the bisection-search to find $\mu \in [0,x^{\mathrm{uppbound}}]$ such that $G^{\prime }\left(\mu \right)=0$.18: **if** $\mu >\min_{i\in I}\left\{x_{i}^{\mathrm{largest}}\right\}$ **then**19:  Set $x^{*}=\min_{i\in I}\left\{x_{i}^{\mathrm{largest}}\right\}$.20: **else**21:  **if** $\mu <\max\{\frac{1}{T^{\max}},\frac{\alpha }{\theta }\}$ **then**22:   Set $x^{*}=\max\left\{\frac{1}{T^{\max}},\frac{\alpha }{\theta }\right\}$.23:  **else**24:   Set $x^{*}=\mu$.25:  **end if**26: **end if**27:
**else**
28: Set $x^{*}=\max\left\{\frac{1}{T^{\max}},\frac{\alpha }{\theta }\right\}$.29:
**end if**
30:$V_{\theta }=G\left(x^{*}\right)$.31:**Output**: $V_{\theta }$ and $t_{\theta }=\frac{1}{x^{*}}$.


Until now, we have proposed Algorithm 1 that solves Problem (P2-Sub) and obtained the value of $V_{\theta }$ for the given value of θ.

### 3.2. Proposed Algorithm to Solve Problem (P2-Top)

After obtaining $V_{\theta }$ for the given θ, we continue to solve the original Problem (P2-Top), i.e., finding the minimum value of θ (which is denoted by $\theta^{*}$) such that $V_{\theta }\leq 0$. As we have explained before, $V_{\theta }$ decreases in θ, which thus enabled us to use the bisection search method to find $\theta^{*}$ [26]. The details are shown in our proposed Algorithm 2.

In our Algorithm 2, for the currently given $\theta^{\mathrm{cur}}$, we invoke Algorithm 1 (as a subroutine) to obtain the value of $V_{\theta^{\mathrm{cur}}}$ (i.e., Step 4). If $V_{\theta^{\mathrm{cur}}}\leq 0$, and then we use the bisection method to further reduce $\theta^{\mathrm{cur}}$ (i.e., Step 6). Otherwise ($V_{\theta^{\mathrm{cur}}}<0$), we use the bisection method to increase $\theta^{\mathrm{cur}}$ (i.e., Step 8). The operations of the bisection search continued until we reach convergence.

**Algorithm 2** To solve Problem (P2-Top) and find $\theta^{*}$.
1:**Initialization:** Set $\theta^{\mathrm{uppbound}}$ as a sufficiently large number and $\theta^{\mathrm{lowbound}}=0$. Set the tolerable computation error, ϵ.2:
**while** $|\theta^{\mathrm{uppbound}}-\theta^{\mathrm{lowbound}}|>\epsilon$ **do**
3: Set $\theta^{\mathrm{cur}}=\frac{\theta^{\mathrm{uppbound}}+\theta^{\mathrm{lowbound}}}{2}$.4: Given $\theta^{\mathrm{cur}}$, use Algorithm 1 to obtain $V_{\theta^{\mathrm{cur}}}$.5: **if** $V_{\theta^{\mathrm{cur}}}\leq 0$ **then**6:  Set $\theta^{\mathrm{uppbound}}=\theta^{\mathrm{cur}}$.7: **else**8:  Set $\theta^{\mathrm{lowbound}}=\theta^{\mathrm{cur}}$.9: **end if**10:
**end while**
11:Set $\theta^{*}=\theta^{\mathrm{cur}}$12:Given $\theta^{*}$, use Algorithm 1 to obtain $t^{*}=t_{\theta^{*}}$.13:**Output**: $\theta^{*}=\theta^{\mathrm{cur}}$.


Until now, we have proposed the Algorithm 2 that solves Problem (P2-Top) and obtained the optimal value of $\theta^{*}$. Notice that based on (21), the minimum total cost for resource consumption $O^{m}=\theta^{*}$ is obtained for the *m*-th decoding-order, $\pi^{m}$, which thus solves the original Problem (P1-*m*).

### 3.3. Numerical Results for Algorithms 1 and 2 to Solve Problem (P1-*m*)

In this subsection, we firstly evaluate the performance of our proposed Algorithms 1 and 2 to solve Problem (P1-*m*). To this end, we set up a scenario in which the AP is located at (0 m , 0 m), and the group of STs are uniformly distributed within a plane whose central is the AP and the radius is 100 m. We use the same method used by reference [27] to model the channel power gains from the STs to the AP. We set each ST’s energy budget as $E_{i}^{\max}=4$ J, and we set $T^{\max}$ = 1 sec. For simplicity, we set the cost coefficients as α=1 and β=1. Other parameter settings will be provided when needed.

To test the performance of our proposed Algorithm 2 (and Algorithm 1), we use a fixed decoding-order for the STs I, i.e., the STs executed the SIC according to descent order of the channel power gains ${\left\{g_{iA}\right\}}_{i\in I}$. Here, we emphasize that Algorithms 1 and 2 are applicable to an arbitrary decoding-order of the STs. In the next section, we further show the performance of our proposed Algorithm 2 (and Algorithm 1) for different decoding-orders.

**Figure 3:**
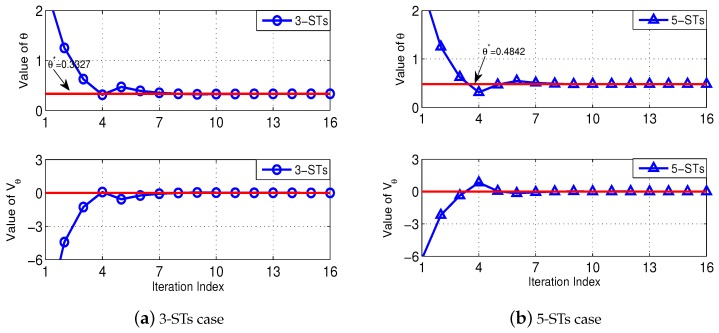
Illustration of the operations of Algorithm 2.

Figure 3 illustrates the rationale of our Algorithm 2 to solve Problem (P2-Top). Specifically, we test two cases, i.e., a 3-ST case and a 5-ST case. In both cases, the locations and channel power gains of the STs are generated as described before, and we set each ST’s $s_{i}^{\mathrm{req}}$ to be uniformly distributed within [2,8] Mbits, and we use a channel bandwidth of W=8 MHz. In both Figure 3a,b, the top-subplots show the convergence of θ when executing the Algorithm 2, and the bottom-subplots show the corresponding convergence of $V_{\theta }$. As illustrated before, Algorithm 2 essentially executes a bisection search on θ, with the objective of finding the minimum value of θ such that $V_{\theta }\leq 0$. Therefore, we observe from the bottom-subplots that the value of $V_{\theta }$ gradually converges to zero (as indicated by the red-solid line), and correspondingly, in the top-subplots we observe that the value of θ converges to $\theta^{*}$ (i.e., the minimum total cost) when $V_{\theta }$ is approaching zero.

**Figure 4:**
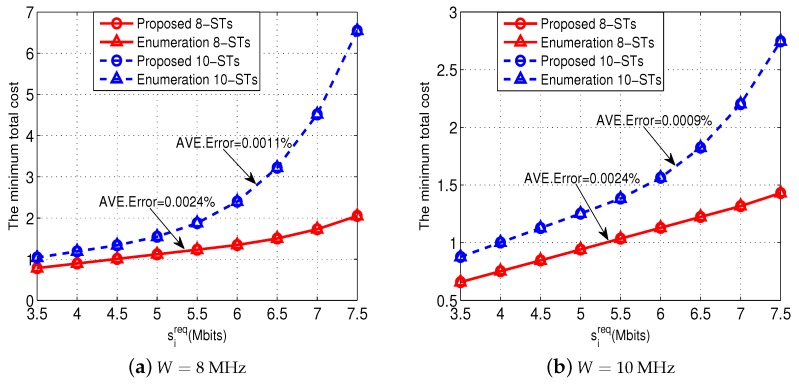
Effectiveness of the Algorithms 1 and 2 in solving Problem (P1-*m*).

Figure 4 shows the effectiveness of our proposed Algorithms 1 and 2 to solve Problem (P1-*m*). For the purpose of comparison, we use an enumeration method to solve Problem (P1-*m*) directly and obtain the minimum total cost as a benchmark. Figure 4a shows the comparison when W=8 MHz, and Figure 4b shows the comparison when W=10 MHz. Notice that in both the 8-ST and 10-ST cases, the locations and the channel power gains of the STs are randomly generated. All the results show that our Algorithm 2 achieves the minimum total costs which are very close to those obtained by the enumeration method (with almost negligible error). These results validate the effectiveness of our proposed Algorithms 1 and 2.

## 4. Proposed Algorithm to Find Optimal Decoding-Order

In Section 3 before, we solve Problem (P1-*m*) and obtained the minimum total cost, $Q^{m}$, for the *m*-th decoding-order $\pi^{m}$. We next continue to solve Problem (P1-Order) in Equation (9) to find the optimal decoding-order which yields the globally minimum cost, i.e., $\min_{m=1,2,\ldots ,I!}Q^{m}$.

As we have described before, a benchmark approach to find the optimal decoding-order was done to enumerate all the possible decoding-orders $\pi^{m}$ for m=1,2,…,I! (e.g., enumerating the six decoding-orders for the 3-ST as shown in Figure 2). However, enumerating all the decoding-orders is computational-prohibited, especially when the number of STs is large. For instance, for the case of 11 STs, the total number of the decoding-orders is larger than 39 million.

To tackle this difficulty, we propose a computation-efficient algorithm (i.e., Algorithm 3) to find the optimal decoding-order. Our Algorithm 3 works as follows.

- We first initialize the currently available STs as $I^{\mathrm{cur}}=I$ and the current best decoding-order (i.e., CBS) as CBS=∅.
- In each round of iteration, we select one ST in $I^{\mathrm{cur}}$ and determine its index in the current best decoding-order CBS, with the objective of minimizing the total cost for resource utilization after including this selected ST into CBS. To this end, we enumerate the currently available STs in $I^{\mathrm{cur}}$. For each selected ST (let us say ST *m*, i.e., the *m*-th ST in $I^{\mathrm{cur}}$), we further enumerate all the possible indexes for decoding based on CBS. Specifically, if h=0, it means that we place ST *m* in front of the first ST in CBS. If h=|CBS|, it means that we place ST *m* after the *h*-th ST in CBS. Otherwise, it means that we place ST *m* between the *h*-th ST in CBS and the h+1-th ST in CBS.
- Given the currently constructed decoding-order $I^{\mathrm{cur}.\mathrm{test}}$, we use our proposed Algorithm 2 to compute the minimum total cost for resource utilization denoted by $\theta^{\mathrm{cur},\mathrm{test}}$. If $\theta^{\mathrm{cur},\mathrm{test}}<\mathrm{CBV}$ (where CBV denotes the currently minimum total cost when enumerating the STs in $I^{\mathrm{cur}}$ and the possible indices based on CBS), then we update $\mathrm{CBV}=\theta^{\mathrm{cur},\mathrm{test}}$ and record the current best decoding-order $\mathrm{CBS}=I^{\mathrm{cur}.\mathrm{test}}$. Finally, we update $I^{\mathrm{cur}}=I^{\mathrm{all}}\backslash \mathrm{CBS}$, and then continue the next round of iteration.

Notice that for the sake of easy presentation, in Algorithm 3, we use $|I^{\mathrm{cur}}|$ to denote the cardinality of set $I^{\mathrm{cur}}$, and use $I^{\mathrm{cur}}\left(m\right)$ to denote the *m*-th element in set $I^{\mathrm{cur}}$, and use $I^{\mathrm{cur}}\left(m:n\right)$ to denote the subset of $I^{\mathrm{cur}}$, i.e., from the *m*-th element to the *n*-th element in $I^{\mathrm{cur}}$.

**Figure 5:**
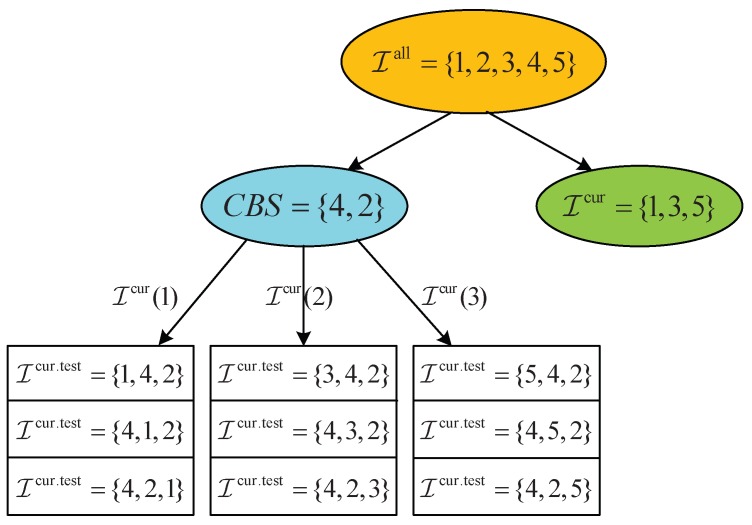
An illustrative example when executing Algorithm 3.

Figure 5 provides an illustrative example when executing our proposed Algorithm 3. Specifically, we consider a 5-ST case, i.e., I={1,2,3,4,5}. In the current iteration, shown in Figure 5, the current best decoding-order is CBS={4,2} and the currently available STs are $I^{\mathrm{cur}}=\left\{1,3,5\right\}$. Thus, when we enumerate the STs in $I^{\mathrm{cur}}$ in the current round of iteration, there are three possible STs, i.e., $I^{\mathrm{cur}}\left(1\right)=1$, $I^{\mathrm{cur}}\left(2\right)=3$, and $I^{\mathrm{cur}}\left(3\right)=5$. Furthermore, when we select the first ST in $I^{\mathrm{cur}}$ (i.e., ST 1), there are three possible indices in CBS to place the ST, which correspond to $I^{\mathrm{cur},\mathrm{test}}=\left\{1,4,2\right\}$, $I^{\mathrm{cur},\mathrm{test}}=\left\{4,1,2\right\}$, and $I^{\mathrm{cur},\mathrm{test}}=\left\{4,2,1\right\}$.

In summary, in our proposed Algorithm 3, we try to strike a balance between achieving optimality and reducing the complexity. Specifically, our proposed Algorithm 3 uses an iterative manner to determine the optimal decoding-order for the STs one-by-one. In each round of iteration, given the currently ordered subgroup of the STs (in which the decoding-orders of the STs have been determined), we optimally select a new ST from the remaining unordered STs and place this new ST into the currently ordered subgroup. To reduce the complexity, we do not change the decoding-orders of the STs in the currently ordered subgroup and aim to place the new ST at the most appropriate index such that we can achieve the minimum overall cost after including this newly selected ST into the currently ordered subgroup. Such an approach continues until we finish determining the decoding indices for all STs in I. Notice that our Algorithm 3 requires the total number of iterations to be equal to $\sum_{i=1}^{I}\left(I-\left(i-1\right)\right)*i$, which is in order of $O\left(I^{2}\right)$. Thus, compared with enumerating all possible I! decoding-orders, our Algorithm 3 gains a significant advantage in saving the computational complexity, especially when the number of STs is large. Table 1 in the following text verifies this advantage. Notice that since the computation complexity of our Algorithm 3 increases quadratically with the number of the STs, its computation time will become non-negligible when the number of STs is large. To address this issue, a viable approach is to jointly exploit the NOMA and TDMA; namely, we first use TDMA to divide a large number of the STs into several nonoverlapping (small) subgroups, with different subgroups using different nonoverlapping time-slots. Meanwhile, for the STs within the same subgroup, we can use our proposed Algorithm 3 to determine the optimal decoding-order efficiently.
**Algorithm 3** To find the optimal decoding-order.1:**Initialization:** Set $I^{\mathrm{cur}}=I^{\mathrm{all}}=\left\{g_{1A},g_{2A},\ldots ,g_{IA}\right\}$ and set CBS=∅2:**while**$I^{\mathrm{cur}}\neq \emptyset$**do**3: Set CBV is a sufficiently large number.4: **for** $m=1:1:|I^{\mathrm{cur}}|$ **do**5:  **for** h=0:1:|CBS| **do**6:   Set $I^{\mathrm{cur}.\mathrm{test}}=\emptyset$.7:   **if** h==0 **then**8:    $I^{\mathrm{cur}.\mathrm{test}}=\left\{I^{\mathrm{cur}}\left(m\right),CBS\right\}$.9:   **else**10:    $I^{\mathrm{cur}.\mathrm{test}}=\{CBS\left(1:h\right),I^{\mathrm{cur}}\left(m\right),CBS(h+1:|CBS|)\}$.11:   **end if**12:   Given $I^{\mathrm{cur}.\mathrm{test}}$, use Algorithm 2 to compute $\theta^{\mathrm{cur}.\mathrm{test}}$.13:   **if** $\theta^{\mathrm{cur}.\mathrm{test}}<CBV$ **then**14:    Set $CBV=\theta^{\mathrm{cur}.\mathrm{test}}$15:    Set $CBS=I^{\mathrm{cur}.\mathrm{test}}$16:   **end if**17:  **end for**18: **end for**19: Update $I^{\mathrm{cur}}=I^{\mathrm{all}}\backslash CBS$.20:**end while**21:**Output**: $\theta^{*}=CBV$.

**Figure 6:**
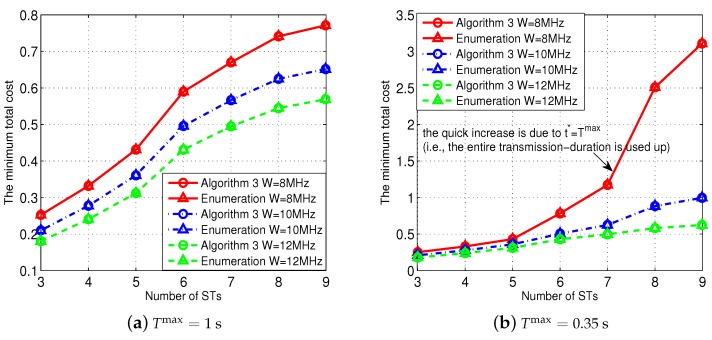
Performance of our proposed NOMA-transmission scheme under different numbers of STs.

We next evaluate the effectiveness of our proposed Algorithm 3 to find the optimal decoding-order, i.e., solving Problem (P1-Order), in Figure 6. Specifically, Figure 6a shows the results when we set $T^{\max}=1$, and Figure 6b shows the results when we set $T^{\max}=0.35$ (meaning that there was a relatively smaller freedom in adjusting the STs’ uplink transmission-duration). As a benchmark for comparison, we enumerate all possible decoding-orders for the group of STs I. As we have explained before, each ST’s data requirement, $s_{i}^{\mathrm{req}}$, was randomly generated according to a uniform distribution within [2,8] Mbits. All comparison results in Figure 6a,b show that our Algorithm 3 can find the optimal decoding-order which is exactly same as the one obtained by the enumeration method, which thus validates the effectiveness of our proposed Algorithm 3.

To further verify the computational efficiency of our Algorithm 3, in Table 1, we compare the computation-time used by our Algorithm 3 with the enumeration method. The results show that the computation time used by the enumeration method increases explosively when the number of the STs increases (which is due to the fact that the total number of possible decoding-orders increases explosively when the number of the STs increases). In contrast, our Algorithm 3 consumes a significantly less computation time compared with the enumeration method. In particular, when the number of the STs large (e.g., larger than 7), the computation time used by our Algorithm 3 is almost negligible compared with the enumeration method.

**Figure 7:**
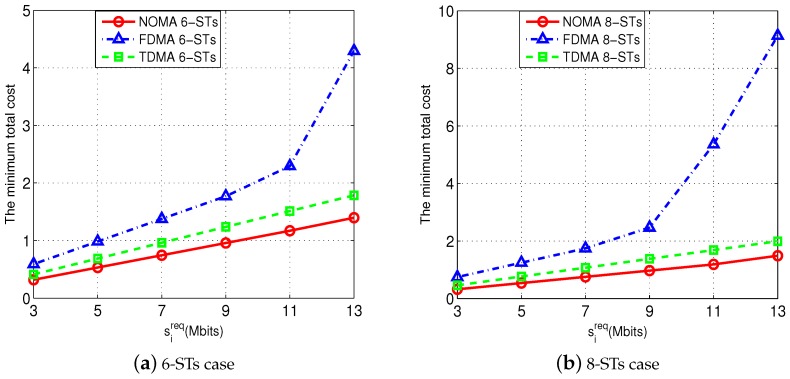
Performance of our proposed NOMA-transmission scheme in comparison with the FDMA scheme and TDMA scheme.

Furthermore, Figure 7 shows the performance advantage of our proposed optimal NOMA transmission scheme in saving the total cost of resource utilization. For the purpose of comparison, we also use two other orthogonal multiple access (OMA) schemes, namely, the FDMA scheme and the TDMA scheme (notice that neither the frequency division multiple access (FDMA) scheme nor the time division multiple access (TDMA) scheme requires to order the STs). We set W=8 MHz, and varied each ST’s $s_{i}^{\mathrm{req}}$ from 3 Mbits to 13 Mbits. We test two cases, namely, the 6-ST case in Figure 7a and the 8-ST case in Figure 7b. It is reasonable to observe that, for all the three schemes, the corresponding total costs increase when each ST *i*’s $s_{i}^{\mathrm{req}}$ increases. Furthermore, the results show that our proposed NOMA-transmission scheme (i.e., the optimal solution obtained by our Algorithm 3) can significantly reduce the total cost, compared with the TDMA scheme and FDMA scheme.

**Figure 8:**
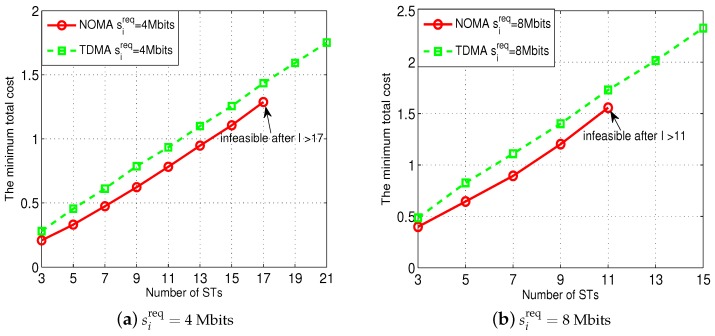
Performance of our proposed NOMA transmission scheme under different numbers of STs. Each point denotes the average result of 100 sets of the random realizations of the STs’ locations.

We finally show the impact of the number of the STs on the performance of our proposed NOMA transmission scheme in Figure 8. In particular, the NOMA enables all STs to transmit to the AP simultaneously. As a result, the co-channel interference among the STs might negatively influence their throughput to the AP especially when the number of the STs is large. The results in Figure 8 verify this intuition. Specifically, as shown in Figure 8a (where each ST’s $s_{i}^{\mathrm{req}}=4$ Mbits) and Figure 8b (where each ST’s $s_{i}^{\mathrm{req}}=8$ Mibts), when the number of the STs increase, the optimal total cost used by our proposed NOMA transmission scheme gradually increases and is always smaller than the cost used by the TDMA scheme. Nevertheless, as shown in Figure 8a, our proposed NOMA transmission scheme becomes infeasible (meaning that the given $T^{\max}$ and ${\left\{E_{i}^{\max}\right\}}_{i\in I}$ cannot satisfy all STs’ ${\left\{s_{i}^{\mathrm{req}}\right\}}_{i\in I}$) when the number of the STs is larger than 17, while the TDMA scheme is always feasible. Figure 8b shows similar infeasible results when using our NOMA transmission scheme if the number of the STs is larger than 11. Such a result essentially stems from the co-channel interference introduced by the NOMA transmission, while there is no co-channel interference in the TDMA scheme.

## 5. Conclusions

In this paper, we investigated the uplink transmission in a sensor network in which a group of STs use NOMA to send their collected data to an AP. We studied joint optimization of the SIC decoding-order, time and power allocations for the STs’ NOMA transmissions, with the objective of minimizing the total cost for resource consumption. Despite the nonconvexity of the formulated joint optimization problem, we proposed efficient algorithms to find the optimal solution. Numerical results validated the effectiveness of our proposed algorithms and the performance advantage of our proposed optimal NOMA transmission for the uplink data collection in sensor networks in comparison with the conventional OMA schemes. In future work, we will take into account cases in which some ST data volumes cannot be satisfied and propose a feasibility-checking algorithm (e.g., to find the maximum number of the STs which are affordable to serve in the uplink NOMA transmission). In addition, since NOMA has been considered to be one of the enabling technologies for future low-latency and ultra-reliable communications (URLLC) [28], we will further investigate the exploitation of NOMA for URLLC and the corresponding optimal resource management.

## Figures and Tables

**Table 1:** Comparison of the computation time (in seconds). The results are obtained on a PC with Intel(R) Core(TM) i5-7400 CPU@3.00 GHz.

W=8 **MHz**	**3-ST**	**4-ST**	**5-ST**	**6-ST**	**7-ST**	**8-ST**	**9-ST**
Proposed	1.7	2.3	2.9	3.5	4	4.6	5.3
Enumeration	1.6	3.5	13.7	65.8	416	2830	16,366
**W=10 MHz**	**3-ST**	**4-ST**	**5-ST**	**6-ST**	**7-ST**	**8-ST**	**9-ST**
Proposed	1.7	2.3	2.9	3.5	4	4.5	5.3
Enumeration	1.5	4.6	18.2	101	562	4099	24,215
**W=12 MHz**	**3-ST**	**4-ST**	**5-ST**	**6-ST**	**7-ST**	**8-ST**	**9-ST**
Proposed	1.7	2.3	2.9	3.5	3.7	4.5	5.5
Enumeration	1.8	5.7	22.3	126	737	5201	31,730
